# Cherokee County Medical Association

**Published:** 1889-07

**Authors:** J. H. Evans, J. N. B. Guinn

**Affiliations:** Secretary; Alto, Texas; President; Alto, Texas


### CHEROKEE COUNTY MEDICAL ASSOCIATION.

ALTO, TEXAS, June 8, 1889.

The Cherokee County Medical Association held its fourth quarterly meeting April 4th, 1889. The President, Dr. J. N. B. Guinn in the chair. The attendance was unusually large and much interest manifested. The President read his address, with such recommendations as he thought proper for the welfare of the Association. It was practical and to the point.

Standing committees were called for, when Dr. L. D. Guinn, chairman of the committee on collective investigation, responded with a report on cholera infantum which was especially interesting to the association. Dr, McCord read a minority report on cholera infantum, which was also interesting and evoked a lively discussion.

Dr. J. H. Evans read a paper on pueperal eclampsia, with clininal reports of three cases. His treatment was in the main, morphine and veratrum viride, which acted like a charm.

The subjects and writers for next meeting are as follows:

Dr. H. P. Clark; subject, cerebro-spinal miningitis.

Dr. J. W. Rogers; subject, fistula in ano.

Dr. C. A. Wade; subject, small-pox.

Dr. J. W. Ramsay; subject, dysentery.

On motion the meeting adjourned, to meet at New Birmingham on the first Thursday in July, 1889,

J. H. EVANS, M. D, Secretary. — J. N. B. GUINN, M. D., President.

